# Dissolution

**Published:** 1885-06

**Authors:** 


### Dissolution.

THE firm of Spencer & Crocker, dealers in dental goods and supplies, has, by mutual consent, been dissolved. The business will be continued at the old stand by Sam'l A. Crocker & Co.

A full supply of the best materials for the dentist's use will be kept. As to the management of the business, it is only necessary to say it will be under the supervision of the indefatigable CROCKER, with the genial Ed. Harmeyer as the cö-efficient.

